# Mass Transfer in Osmotic Dehydration of Kiwiberry: Experimental and Mathematical Modelling Studies

**DOI:** 10.3390/molecules23051236

**Published:** 2018-05-22

**Authors:** Michał Bialik, Artur Wiktor, Piotr Latocha, Ewa Gondek

**Affiliations:** 1 Faculty of Food Sciences, Department of Food Engineering and Process Management, Warsaw University of Life Sciences, Nowoursynowska 159c, 02-776 Warsaw, Poland; michal_bialik@sggw.pl (M.B.); artur_wiktor@sggw.pl (A.W.); 2 Faculty of Horticulture, Biotechnology and Landscape Architecture, Department of Environmental Protection, Warsaw University of Life Sciences, Nowoursynowska 159, 02-776 Warsaw, Poland; piotr_latocha@sggw.pl

**Keywords:** kiwiberry, osmotic dehydration, polyols, water loss, solid gain, mathematical modelling

## Abstract

The aim of this study was to analyze the impact of osmotic solutions and temperature on the osmotic dehydration (OD) of two cultivars of kiwiberry. OD was carried out in sucrose, xylitol and maltitol solutions at 30 °C and 50 °C, respectively. The process of osmotic dehydration was described by the means of water loss (WL), solid gain (SG), weight reduction (WR), and water content changes. Moreover, dehydration was described by mathematical models often used in the literature. The highest WL, WR and SG values were observed for samples treated by xylitol and maltitol at 50 °C. The statistical analysis of the mathematical modelling of the process showed that in most cases, the Peleg’s equation exhibits better fitting for the experimental data.

## 1. Introduction

Food preservation is a process performed in order to slow down spoilage, loss of quality, and improve edibility and nutritional values. This usually involves inhibiting microorganisms growth as well as slowing down chemical reactions, such as oxidation of fats or discoloration caused by enzymatic browning [1]. Continuous interests placed on extending food shelf-life with improving food quality and safety, resulted in the development of “minimal” preservation techniques and adaptation of traditional methods, and the ways they can be combined with other technologies. Osmotic dehydration (OD) is one of the most popular traditional methods, since it can be easily combined with subsequent technological steps [2,3].

OD is a process in which migration of water through a semi permeable membrane, from a lower concentration of solute to a higher concentration, resulting in an equilibrium condition on both sides of membrane [1,4]. This process lowers both water content and water activity of a treated material. Properly applied, it improves retention of color, aroma, flavor and nutrients during further processing or storage [3]. Dehydration often precedes different preservation treatments, because when applied alone it is not sufficient to inhibit the growth of microbiota [2,5].

For OD, different types of agents are used. The most common agents are sugar, salt, and brine in the case of some vegetables [1]. Kinetics of the process depends on osmotic pressure, which is based on molarity and activity coefficient. With the same molarity, osmotic pressure depends only on the osmotic coefficient, which depends, in turn, on the temperature and molarity of a solute [6,7]. Although the goal of this method is to remove water from foods, in reality, it is a two-way mass transfer process [8]. Rate, effectiveness and outcome of this process depend on multiple factors, such as time, temperature, solute and its concentration, and type of dehydrated material [2,5]. Determination of proper parameters can result in obtaining foods that possess desired parameters and quality. As mentioned, OD often precedes further food processing steps, and in most cases, different methods of drying [9]. Public demand for less-caloric food induced the development of foods, replacing sugar with artificial sweeteners and polyols. The most popular osmotic agent is sugar, but it can strongly influence dehydrated products, especially if they often increase sugar content; for example, dried cranberries can contain up to 72.56% sugars [10]. Polyols have strong dehydrating capabilities and a sweetness similar to sucrose, and have low caloric values [11]. Osmotic pre-treatment of microwave-convective dried blueberries resulted in similar properties to those of untreated freeze-dried fruits [12].

Kiwi (*Actinidia chinensis*) is a popular fruit, which is known for its positive nutritional and health values, and is a unique enzyme, which promotes food digestion; that is, actinidine. A similar biochemical composition with the presence of the same enzyme can be found in kiwiberry (*Actinidia arguta*). Kiwiberry fruits are small and weigh between 6 g and 12 g. They have smooth and hairless skin, so they can be eaten whole [13,14,15]. Kiwiberry is rich in vitamin C, minerals, phenolic acids and pigments, and has proven antioxidant and bacteriostatic properties, and they are widely known for their strong enzymatic activity [14]. Untreated fruits can be stored in cold stores up to six weeks, during which, bioactive compounds degrade and nutritional properties diminish. Not all fruits have optimum quality for direct consumption, and can be eaten only after some form of processing. Sustainable development requires establishing methods for managing variety of fruits, which is the reason why different methods of treatment and pre-treatment are still researched.

The aim of this study was to analyze the impact of different osmotic solutions and temperature on the OD of two cultivars of kiwiberry. According to the best knowledge of the authors, this is the first article that deals with this topic in the case of kiwiberry fruits.

## 2. Results and Discussion

### 2.1. Weight Reduction 

**Figure 1:**
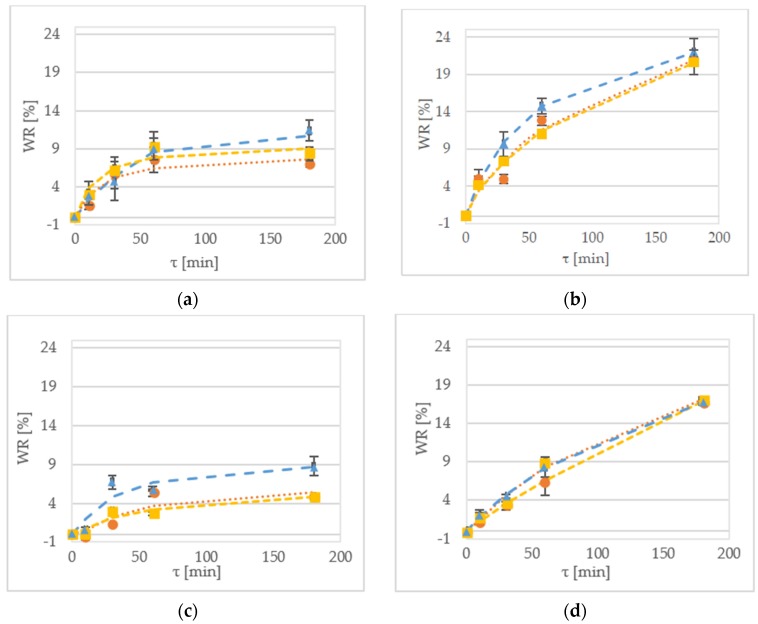
WR kinetics of the ‘Geneva’ cultivar dehydrated at (**a**) 30 °C; and (**b**) 50 °C in xylitol (▲—triangle), maltitol (■—square) and sucrose (●—circle). WR kinetics of the ‘Weiki’ cultivar dehydrated at (**c**) 30 °C; and (**d**) 50 °C in xylitol (▲—triangle), maltitol (■—square) and sucrose (●—circle). Dotted lines represent values obtained from the mathematical modelling.

Weight reduction (WR) denotes the net difference in weight between the initial weight of the sample and the weight of the dehydrated fruit based on the initial sample weight [16]. Figure 1 shows the WR of the samples during the OD of kiwiberry tissue. After 1 h of processing the ‘Geneva’ cultivar at 30 °C, the WR values were equal to 7.5%, 9.4% and 8.9% for the samples treated by sucrose, maltitol and xylitol, respectively (Figure 1a). In comparison, the values obtained for the dehydration of the ‘Weiki’ cultivar were equal to 5.5%, 2.6% and 5.7% for the samples treated by sucrose, maltitol and xylitol, respectively (Figure 1b). For the same process performed at 50 °C, the values obtained for the ‘Geneva’ cultivar were equal to 12.8%, 11.0% and 14.7% for sucrose, maltitol and xylitol, respectively (Figure 1b). For the ‘Weiki’ cultivar, the WR values were 6.5%, 8.9% and 13.6% for the samples treated by sucrose, maltitol and xylitol, respectively (Figure 1d). The ‘Geneva’ cultivar seemed to be more susceptible to OD, so it had higher WR values, which probably resulted from the thinner skin of the ‘Geneva’ fruits, whereas the ‘Weiki’ fruits have a higher endurance against environmental factors. In the case of this experiment, their resistance against mass transfer, which was represented by lower WR values, was diminished after the temperature was raised. The 50 °C tested samples showed similar and comparable results despite using different osmotic solutions (Figure 1c,d). After 180 min of the process carried out at 30 °C, the ‘Geneva’ kiwiberry fruits reached the WR values of 7.0% and 11.3% for sucrose and xylitol, respectively (Figure 1a). The same solutions at 50 °C provided WR values of 20% to 22% for sucrose and xylitol, respectively (Figure 1b). For the ‘Weiki’ cultivar, after 60 min of OD at 30 °C, WR values were 4.7% for sucrose and 8.7% for xylitol, respectively (Figure 1c). Applying high temperature resulted in a maximum WR value of 17.0% for maltitol and 16.8% for xylitol, respectively (Figure 1d).

During the experiment, there was a noticeable slowing down of WR kinetics. Water leaving the tissue lowered osmotic pressure and influenced further processing. Occurrence of this phenomena could be diminished by increasing the ratio of solution to sample mass, which was used in the experiment performed by Kowalski and Mierzwa [3] and Tiroutchelvame et al. [17]. In the study by Arballo et al. [18], researchers suggested that the optimum value for dehydrated cubes of fruits (pumpkin, kiwi, pear) resides between 112 min and 240 min of processing. For the kiwi cubes [18], established by Arballo et al., the WR value equal to 27.8% was obtained when they were in 60% solute of sucrose for 145 min at 30 °C. Comparable observations can be made for kiwiberry halves at 30 °C. In addition, temperature strongly influenced obtained values, which was also confirmed by Alam et al. [19], Yadav et al. [20] and Cichowska et al. [21]. During OD of anola slices researched by Alam et al. [19] at temperatures between 30 °C and 60 °C, the optimum temperature was established at 51 °C. 

In this experiment, xylitol was determined as the most effective osmotic reagent for reaching high WR values (Figure 1). High efficiency of this polyol was confirmed by Mendonça et al. [9,18] and Cichowska et al. [21]. Both authors agree that it is the result of xylitol’s low molecular weight. It is also worth noticing that all the investigated parameters of the OD had significant impacts on the WR values (Table 1). Statistical analysis has also shown interactions between the tested variables (Table 2); a significant for interaction between temperature–time–solution (*p* < 0.05) and a significant for temperature–time (*p* < 0.01) were identified. According to some researchers, WR is mostly influenced by the concentration of the osmotic solution and the temperature [22]. The experiment performed on the kiwifruit slices by Cao et al. [23], showed that the temperature of the process lessened the influence of the other factors or their combinations.

**Figure 2:**
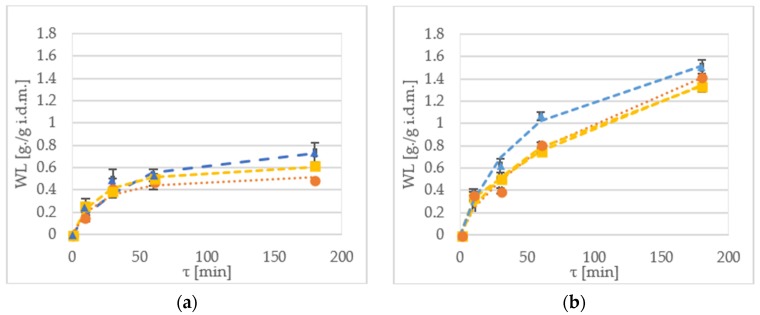

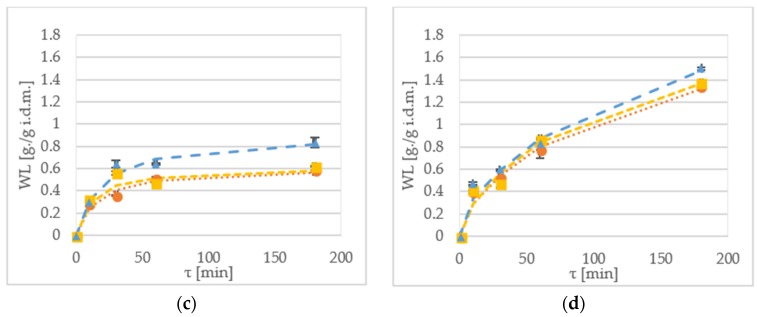
WL kinetics of the ‘Geneva’ cultivar dehydrated at (**a**) 30 °C; and (**b**) 50 °C in xylitol (▲—triangle), maltitol (■—square) and sucrose (●—circle). WL kinetics of the ‘Weiki’ cultivar dehydrated at (**c**) 30 °C; and (**d**) 50 °C in xylitol (▲—triangle), maltitol (■—square) and sucrose (●—circle). Dotted lines represent values obtained from the mathematical modelling.

One of the aims of this study was to determine the best fitting model for OD kinetics. Two popular equations were used and modelled, i.e., Peleg’s [24] and Ade-Omowaye et al. [25]. The results of the statistical evaluation of experimental regression analysis for the WR are presented in Table 3. *CRV* values lower than 20% indicate usefulness of the tested equation for the prediction of the process [26]. Mathematical modelling has shown higher usefulness of the Peleg’s model for the WR prediction. It was the only model that could calculate the expected values for process at 30 °C, although the resulting *CRV* values were too high for practical use. On the other hand, fitting values for 50 °C have given high *R*^2^ values, and low *CRV*, *RMSE* and *χ*^2^ values (Table 3). The Peleg’s equation has two independent variables representing initial mass transfer rate (*K*_1_) and equilibrium moisture content (*K*_2_). The *K*_1_ and *K*_2_ values for the 50 °C process indicated both high transfer and water removal rates (Figure 2a,c). The *K*_1_ values decreased with the increase of the temperature. There was no visible trend for the *K*_2_ values. The usefulness of the Peleg’s model was also confirmed by Arballo et al. [18] when researchers were using this model for predicting the OD of different fruits.

### 2.2. Water Loss 

Water loss (WL) is a parameter that indicates amount of water removed during pre-treatment in relation to initial sample mass [16]. Figure 1 shows water loss of samples during the OD of kiwiberry tissue. In most cases, the WL value for samples processed with xylitol, was higher when compared to the other osmotic solutions. For instance, after 10 min of immersion, the WR value at 30 °C of the ‘Geneva’ cultivar was 0.14 g/g initial dry matter (i.d.m.) and 0.25 g/g i.d.m. for sucrose and maltitol, respectively (Figure 2a). In turn, for the ‘Weiki’ cultivar, the WL reached values of 0.33 g/g i.d.m. for sucrose and 0.37 g/g i.d.m. for xylitol, respectively (Figure 2c).

After 60 min of the process, the highest WL values were obtained for the samples dehydrated at 50 °C, reaching values between 1.06 g/g i.d.m. for the ‘Geneva’ cultivar (Figure 2b), and 0.87 g/g i.d.m. for the ‘Weiki’ cultivar (Figure 2d), both dehydrated in xylitol. In the case of the 30 °C treatment in xylitol, the highest WL values were obtained equal to 0.53 g/g i.d.m. for the ‘Geneva’ cultivar (Figure 2a) and 0.64 g/g i.d.m. for the ‘Weiki’ cultivar (Figure 2c). Prolonging the process up to 180 min allowed further water removal. During this experiment, the highest WL values for both the ‘Geneva’ and ‘Weiki’ cultivars, were obtained from the samples processed with xylitol after 180 min of the OD at 50 °C, which reached up to 1.5 g/g i.d.m. (Figure 2b,d). The lowest experimental values of the WL were achieved for materials kept at 30 °C in sucrose, which were 0.48 g/g i.d.m. and 0.57 g/g i.d.m. for the ‘Geneva’ and ‘Weiki’ cultivars, respectively (Figure 2a,c).

The established WL values suggest that 60 min OD of kiwiberry at 30 °C allows to achieve results similar to 30 min immersion at 50 °C. As expected, the maximum WL values were reached in the case of the longest processing time (180 min) performed at the highest temperature (50 °C). Such high temperature, although it is beneficial for OD kinetics, can influence biochemical properties of the material [27]. Statistical analysis showed significant differences in applied temperatures, osmotic solutions and process durations. There was no significant difference between the tested cultivars (Table 4). There were a few significant (*p* < 0.05) interactions between the tested variables (Table 2) for cultivar–time, temperature–time–solution, and very strong interaction (*p* < 0.01) for variables of temperature–time and time–solution.

Difference in osmotic pressure between solution and tissue is the driving force of the dehydration. Calculated ideal osmotic pressures, based on the equation given by Held et al. [7] with the used solutes at 30 °C, were 4418.13 kPa, 4392.33 kPa and 9939.70 kPa for sucrose, maltitol and xylitol, respectively. At 50 °C, the established osmotic pressures were 4709.61 kPa, 4682.12 kPa and 10595.46 kPa for sucrose, maltitol and xylitol, respectively. During the process, water migrates to the solution, which lowers osmotic agents density and as a result, slows down OD [1,28]. High initial WL ratio after the first 2 h of OD, was also observed by Panagiotou et al*.* [29], who were dehydrating apples, bananas and kiwis using 40% sucrose at 40 °C. Similar observations were also made by Arballo et al. [18], where scientists dehydrated pumpkins, kiwis and pears. They have noticed that the time of the process influences the outcome of the dehydration during the first 4 h of the process. In this experiment, similar observations can be made for samples immersed in samples at 30 °C. For kiwiberry treated at 50 °C, it should be taken into consideration that lengthening the process up to at least 4 h establishes plateau values. With the increased temperature, the viscosity of the osmotic reagent decreases, and as a result, the mass transfer improves [20]. The influence of temperature on OD was confirmed by Ciurzyńska et al*.* [30], in the OD of apples at temperatures of 40 °C and 60 °C. In this experiment, xylitol was determined as the most effective osmotic reagent (Figure 2). It is also worth noticing that difference in dehydration effectiveness between sucrose and maltitol turned out to be statistically insignificant (Table 4). This was caused by the molecular weight of the solutes used. Lower molecular weight yields higher osmotic pressure (maltitol 344.31 g/mol, sucrose 342.3 g/mol and xylitol 152.15 g/mol). 

The Peleg’s modelling of the WL was effective in almost all of the tested combinations of the used variables (Table 5). Goodness of fit for this model has given high *R*^2^ values, and low *CRV*, *RMSE* and *χ*^2^, which is expected for applicable models. High goodness of fit of this model for the OD was also reported by Yadav and Singh [1] and Cichowska et al*.* [21].

The Peleg’s equation has two independent variables, where *K*_1_ represents initial mass transfer rate and *K*_2_ describes equilibrium moisture content. As it was aforementioned, low *K*_1_ and *K*_2_ values indicate both high mass transfer and high water removal rates [21,31]. Measured WL values suggest lower effectiveness of sucrose and maltitol, in comparison to xylitol carried out at 30 °C (Figure 1a,c). Statistical analysis has shown that these two osmotic agents belong to the same homogeneous group (Table 3). This could be explained by similar and high molecular weight of these solutions; such observations were also reported by Mendonça et al*.* [11]. Molecular weight could have also influenced the *K*_1_ values for the OD at 30 °C; such phenomena were also reported by other researchers [28]. In addition, it could be explained by similar osmotic coefficient values, and should be further explored in the future experiments.

### 2.3. Solid Gain 

**Figure 3:**
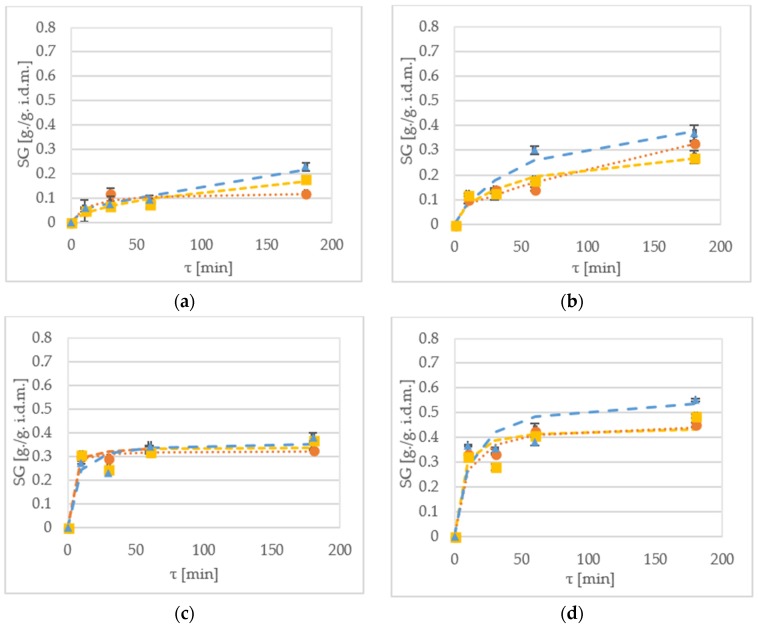
SG kinetics of the ‘Geneva’ cultivar dehydrated at (**a**) 30 °C; and (**b**) 50 °C in xylitol (▲—triangle), maltitol (■—square) and sucrose (●—circle). SG kinetics of. the ‘Weiki’ cultivar dehydrated at (**c**) 30 °C and (**d**) 50 °C in xylitol (▲—triangle), maltitol (■—square) and sucrose (●—circle). Dotted lines represent values obtained from the mathematical modelling.

Solid gain (SG) is a parameter which indicates amount of soluble solids that are incorporated into the sample during dehydration [16]. Figure 3 shows SG of samples during the OD of kiwiberry halves. After 60 min OD of the ‘Geneva’ cultivar at 30 °C, the SG value were 0.08 g/g i.d.m., 0.07 g/g i.d.m. and 0.09 g/g i.d.m. for samples treated by sucrose, maltitol and xylitol, respectively (Figure 3a). The ‘Weiki’ samples were more prone to display higher SG values, which were 0.31 g/g i.d.m., 0.32 g/g i.d.m. and 0.34 g/g i.d.m. for sucrose, maltitol and xylitol, respectively (Figure 3c). When the process was performed at 50 °C, the SG values obtained for the ‘Geneva’ cultivar were 0.14 g/g i.d.m., 0.19 g/g i.d.m. and 0.3 g/g i.d.m. for sucrose, maltitol and xylitol, respectively (Figure 3b). For the ‘Weiki’ cultivar treated at 50 °C, the SG values were 0.43 g/g i.d.m., 0.41 g/g i.d.m. and 0.31 g/g i.d.m. for sucrose, maltitol and xylitol, respectively (Figure 3d). In both temperatures, the ‘Geneva’ cultivar seemed to be more efficient in the SG. As it was mentioned earlier, the dehydration at 50 °C displays the similar effectiveness despite using different osmotic solutions. On the other hand, the 30 °C processing shows larger SG differences between the used solutes. Comparable results for the 60 min dehydration at 50 °C were achieved after 180 min of the OD at 30 °C. Similar observations were also made for shorter processing time. For instance, after 10 min of the immersion at 30 °C, the SG values were similar to the 60 min process at 30 °C.

During the experiment performed by Chiu et al. [32] and Lee et al*.* [33], researchers noticed that SG values are strongly influenced by temperature during the OD of Terung Asam and red algae, respectively. This was also the case during kiwiberry dehydration. The SG values after 180 min dehydration were 25–50% higher when the temperature was raised by 20 °C. According to Lazarides et al. [34], higher temperature promotes faster water loss by plasticizing of cell membranes, increasing water diffusion and lowering viscosity of the osmotic medium. The difference in the SG ratios between the tested cultivars can be explained by the difference in strengths of cell membranes. SG is strongly influenced by ratios of solute to mass [17,32]. Although some researchers suggest the increase of this ratio [3], results of different studies focused on effectiveness and quality of final product [17] claim that a ratio of 5:1 is the optimized value from industrial and scientific perspectives. In this experiment, a ratio of 4:1 was used, which is a well-established experimental standard. Statistical analysis had shown that all the tested parameters had a statistically significant impact on the SG. However, it is worth mentioning that there was no statistical difference between 10 min and 30 min dehydration and between maltitol and sucrose (Table 6).

There were a few significant (*p* < 0.05) interactions between the tested variables (Table 2) for cultivar-solution and very strong interaction (*p* < 0.01) for cultivar-time, temperature-time, time-solution, cultivar-temperature-solution. In comparison to the other variables (WR and WL), SG interactions between variables were more often strongly influenced by osmotic solution.

The Peleg’s mathematical model has shown its high usefulness for the SG prediction (Table 7). The *CRV* values for 30 °C were often exceeding the critical value (20%), but they gives high goodness of fit at 50 °C OD. In most cases, fitting values for 50 °C have provided high *R*^2^ values, and low *CRV*, *RMSE* and *χ*^2^ values. The usefulness of this model was also confirmed by Arballo et al. [15] and Cichowska et al. [21]. No visible trend was established for the K_1_ values of SG for the “Geneva” cultivar. For the “Weiki” cultivar, there was a noticeable reduction in the K_1_ value when the temperature was increased. The impact of the applied temperature on K_1_ values was also reported by Ganjloo et al. [31]. This requires further analysis in future experiments.

## 3. Materials and Methods 

### 3.1. Sample Preparation

The study was performed using two cultivars of kiwiberry: (*Actinidia arguta*) ‘Geneva’ and ‘Weiki’. Plants grew on the commercial orchard under the supervision of the Environmental Protection Department, Warsaw University of Life Sciences (SGGW), Poland. Fruits were collected before reaching eating maturity stage (soft). The material was stored in darkness at 1 °C at a humidity of 90% until it was ripe. Before each experiment, kiwiberry fruits were removed from the storage and left to equilibrate to room temperature (20 ± 1 °C), washed (with tap water) and dried. Ripe fruits were weighed, and the dry matter was determined according to AOAC 920.151 norm [35].

### 3.2. Dehydration Procedure

Before the dehydration process, fruits were cut in halves and weighed. Dehydration was performed using 60% *w*/*w* solutes of sucrose, xylitol and maltitol, respectively. The concentration of these solutes was established during preliminary experiments, and it was based on the results presented in the literature data [18,26]. The OD was executed in a precision hot water bath for 10 min, 30 min, 60 min and 180 min, respectively, with an agitation speed of 1 Hz and water temperature at 30 °C and 50 °C (±1 °C). For each experiment, 22 g (±2.5 g) of kiwiberry halves were put into 250 mL beakers containing 80 mL of osmotic solutions. After reaching the setup dehydration time, the material was removed from the bath, washed using 200 g distilled water, and gently placed on filtration paper to remove liquid residue. Samples were weighed and dry matter was determined. Each osmotic dehydration run was repeated twice.

### 3.3. Analytical Methods

#### 3.3.1. Mathematical Modeling

WR, WL and SG, which are the parameters describing osmotic dehydration process, were calculated according to the following equations:

(1) $$WR=\;\frac{m_{0}\;-\;m_{\tau }}{m_{0}}\;\cdot 100\%$$

(2) $$WL=\;\frac{m_{o}\cdot \;\left(1-\;d.m._{o}\right)-\;m_{\tau }\cdot \left(1-d.m._{t}\right)}{m_{0}\cdot d.m._{0}}$$

(3) $$SG=\frac{\left(m_{t}\cdot d.m._{t}-m_{0}\cdot d.m._{0}\right)}{m_{0}\cdot d.m._{0}}$$

To describe the kinetics of the OD, the following modified equations proposed by Ade-Omowaye et al. [25] and model proposed by Peleg [24] were employed to fit the experimental results. 

WR, WL and SG data (Equations (1)–(3)) for the Ade-Omowaye et al. model were fitted as:

(4) $$WR=\;K_{m}\tau^{0.5}$$

(5) $$WL=K_{\omega }\tau^{0.5}$$

(6) $$SG=\;K_{s}\tau^{0.5}$$

For the Peleg’s model:

(7) $$Y=Y_{0}\pm \frac{\tau }{K_{1}+K_{2}\tau }$$

Fitting of the mathematical functions was performed using Table Curve 2D v5.01 software (SYSTAT Software, Inc., Chicago, IL, USA). The coefficient of determination (*R*^2^) (Equation (11)), the reduced chi-squared statistic (*χ*^2^) (Equation (9)), the root mean square error (*RMSE*) (Equation (8)), and the coefficient of residual variation (*CRV*) (Equation (10)) were used to evaluate the goodness of fit of tested models:

(8) $$RMSE=\;\sqrt{\frac{\Sigma_{i=1}^{N}{\left(MR_{i,p}-\;MR_{i,e}\right)}^{2}}{N}}$$

(9) $$\chi^{2}=\frac{\Sigma_{i=1}^{N}{\left(MR_{i,p}-\;MR_{i,e}\right)}^{2}}{N-n}$$

(10) $$CRV=100\%\;\cdot \frac{\sqrt{\chi^{2}}}{Y}$$

(11) $$R^{2}=\frac{\Sigma_{i=1}^{N}{\left(MR_{i,p}-\;MR_{p}\right)}^{2}}{\Sigma_{i=1}^{N}{\left(MR_{i,e}-\;MR_{p}\right)}^{2}}$$

The high *R*^2^ values and the low *χ*^2^ and *RMSE* values indicate that the model fits well to the experimental data. The values of *CRV* less than 20% indicate that the model can be used for predictions.

#### 3.3.2. Statistical Analysis

The statistical software Statgraphics Plus version 18 (StatPoint), and Excel 2015 (Microsoft) were used for data analysis. The multifactorial analysis of variance (MANOVA) was performed with a significance level of α = 0.05. In the case of significant associations, post-hoc Tukey’s test was performed.

## 4. Conclusions

The maltitol, the xylitol and the sucrose are the effective osmotic agents for dehydration of kiwiberry. Both used polyols (xylitol, maltitol) can be considered as an effective and less-caloric alternative to the commonly used sucrose. For the tested concentration (60%), the most effective solution was xylitol. For the OD process at 30 °C, the WR, WL and SG parameters were similar for maltitol and sucrose. The best fitting model for the mass transfer modelling was the Peleg’s equation. Increased temperature resulted in an increased initial rate of mass transfer and improved overall effectiveness of the process.

## Figures and Tables

**Table 1:** The influence of osmotic pre-treatment in different solutions, and time on WR.

Factor		*p*-Value	Contrast	+/− Limits	Difference
Cultivar	Geneva ^a^	<0.001 *	Geneva–Weiki	0.006	0.034 *
	Weiki ^b^				
Temperature	30 °C ^a^	<0.001 *	30 °C–50 °C	−0.044	−0.005 *
	50 °C ^b^				
Time	10 min ^a^	<0.001 *	10 min–60 min	0.008	−0.062 *
			10 min–30 min	0.008	−0.028 *
			10 min–180 min	0.008	−0.112 *
	30 min ^b^		60 min–30 min	0.008	0.033 *
	1 h ^c^		60 min–180 min	0.008	−0.050 *
	3 h ^d^		30 min–180 min	0.008	−0.084 *
Osmotic solution	Sucrose ^a^	<0.001 *	Xylitol–Maltitol	0.007	0.014 *
	Maltitol ^a^		Xylitol–Sucrose	0.007	0.019 *
	Xylitol ^b^		Maltitol–Sucrose	0.008	0.004

Note: Means within columns, sharing the same superscript, are not significantly different from each other (Tukey’s HSD, *p* < 0.05). * denotes a statistically significant difference.

**Table 2:** Interactions between tested variables for WR, WL and SG expressed by *p*-values.

	Interaction									
	AB	AC	AD	BC	BD	CD	ABC	ABD	ACD	BCD
**WR**	0.148	0.059	0.848	<0.001 *	0.311	0.098	0.938	0.026	0.465	0.022 *
**WL**	0.335	0.009 *	0.304	<0.001 *	0.210	<0.001 *	0.492	0.339	0.074	0.002 *
**SG**	0.554	0.007 *	0.019 *	<0.001 *	0.388	<0.001 *	0.067	0.005 *	0.1898	0.452

A—cultivar, B—temperature, C—time, D—osmotic solution.

**Table 3:** Values of *RMSE*, *χ*^2^, *R*^2^ and *CRV* of best fitting mathematical model of WR.

Cultivar	Temperature	Solution	Model	*K*_1_	*K*_2_	*K*_m_	*RMSE*	*χ* ^2^	*CRV* (%)	*R* ^2^
Geneva	30 °C	sucrose	Ade-Omowaye	-	-	0.006	0.019	0.001	40.89	0.507
			Peleg	11.171	−0.006	-	0.012	0.001	25.11	0.814
		maltitol	Ade-Omowaye	-	-	-	-	-	-	-
			Peleg	9.839	−0.004	-	0.012	0.001	20.30	0.851
		xylitol	Ade-Omowaye	-	-	-	-	-	-	-
			Peleg	8.468	0.003	-	0.017	0.001	21.20	0.896
	50 °C	sucrose	Ade-Omowaye	-	-	-	-	-	-	-
			Peleg	2.780	0.009	-	0.015	0.000	17.35	0.950
		maltitol	Ade-Omowaye	-	-	-	-	-	-	-
			Peleg	2.881	0.011	-	0.008	0.000	9.10	0.985
		xylitol	Ade-Omowaye	-	-	-	-	-	-	-
			Peleg	3.468	0.000	-	0.009	0.000	8.82	0.982
Weiki	30 °C	sucrose	Ade-Omowaye	-	-	-	-	-	-	-
			Peleg	12.525	−0.009	-	0.011	0.000	49.16	0.763
		maltitol	Ade-Omowaye	-	-	-	-	-	-	-
			Peleg	14.953	−0.004	-	0.006	0.000	27.75	0.886
		xylitol	Ade-Omowaye	-	-	-	-	-	-	-
			Peleg	9.001	−0.008	-	0.013	0.000	29.75	0.838
	50 °C	sucrose	Ade-Omowaye	-	-	-	-	-	-	-
			Peleg	1.193	0.001	-	0.006	0.000	10.73	0.989
		maltitol	Ade-Omowaye	-	-	-	-	-	-	-
			Peleg	2.776	−0.002	-	0.007	0.000	10.75	0.986
		xylitol	Ade-Omowaye	-	-	-	-	-	-	-
			Peleg	2.834	0.002	-	0.004	0.000	7.26	0.993

“-” denotes values which cannot be calculated using this prediction model.

**Table 4:** The influence of osmotic pre-treatment in different solutions, and time on WL (g/g i.d.m.).

Factor		*p*-Value	Contrast	+/− Limits	Difference
Cultivar	Geneva ^a^	0.187	Geneva–Weiki	−0.018	0.027
	Weiki ^a^				
Temperature	30 °C ^a^	<0.001 *	30 °C–50 °C	0.027	−0.312 *
	50 °C ^b^				
Time	10 min ^a^	<0.001 *	10 min–60 min	0.039	−0.362 *
			10 min–30 min	0.039	−0.147 *
			10 min–180 min	0.039	−0.733 *
	30 min ^b^		60 min–30 min	0.039	0.215 *
	1 h ^c^		60 min–180 min	0.039	−0.371 *
	3 h ^d^		30 min–180 min	0.039	−0.586 *
Osmotic solution	Sucrose ^a^	<0.001 *	Xylitol–Maltitol	0.034	0.122 *
	Maltitol ^a^		Xylitol–Sucrose	0.034	0.151 *
	Xylitol ^b^		Maltitol–Sucrose	0.033	0.028

Note: Means within columns, sharing the same superscript, are not significantly different from each other (Tukey’s HSD, *p* < 0.05). * denotes a statistically significant difference.

**Table 5:** Values of *RMSE*, *χ*^2^, *R*^2^ and *CRV* of best fitting mathematical model of WL.

Cultivar	Temperature	Solution	Model	*K*_1_	*K*_2_	*K*_ω_	*RMSE*	*χ* ^2^	*CRV* (%)	*R* ^2^
Geneva	30 °C	sucrose	Ade-Omowaye	-	-	0.043	0.119	0.016	35.91	0.538
			Peleg	1.711	−0.028	-	0.056	0.003	16.91	0.897
		maltitol	Ade-Omowaye	-	-	0.052	0.098	0.010	25.01	0.735
			Peleg	1.520	0.012	-	0.037	0.001	9.51	0.961
		xylitol	Ade-Omowaye	-	-	0.079	0.081	0.008	17.87	0.948
			Peleg	1.448	0.013	-	0.082	0.009	11.83	0.959
	50 °C	sucrose	Ade-Omowaye	-	-	0.101	0.079	0.008	13.49	0.967
			Peleg	0.402	0.100	-	0.077	0.007	13.06	0.969
		maltitol	Ade-Omowaye	-	-	0.099	0.029	0.001	5.02	0.995
			Peleg	0.484	0.102	-	0.054	0.004	9.22	0.982
		xylitol	Ade-Omowaye	-	-	0.118	0.082	0.008	11.64	0.969
			Peleg	0.507	0.036	-	0.056	0.004	7.93	0.986
Weiki	30 °C	sucrose	Ade-Omowaye	-	-	0.052	0.095	0.011	27.75	0.663
			Peleg	1.681	0.016	-	0.031	0.001	9.04	0.964
		maltitol	Ade-Omowaye	-	-	0.055	0.104	0.013	28.53	0.618
			Peleg	1.645	0.010	-	0.034	0.001	9.32	0.959
		xylitol	Ade-Omowaye	-	-	0.073	0.120	0.018	25.45	0.761
			Peleg	1.104	−0.002	-	0.035	0.001	7.35	0.980
	50 °C	sucrose	Ade-Omowaye	-	-	0.099	0.045	0.002	7.57	0.986
			Peleg	0.517	0.135	-	0.070	0.006	11.78	0.967
		maltitol	Ade-Omowaye	-	-	0.103	0.067	0.005	10.81	0.973
			Peleg	0.510	0.113	-	0.079	0.007	12.78	0.963
		xylitol	Ade-Omowaye	-	-	0.111	0.060	0.004	9.03	0.980
			Peleg	0.426	0.186	-	0.089	0.010	13.36	0.957

“-” denotes values which cannot be calculated using this prediction model.

**Table 6:** The influence of osmotic pre-treatment in different solutions, and time on SG [g/g i.d.m.].

Factor		*p*-Value	Contrast	+/− Limits	Difference
Cultivar	Geneva ^a^	<0.001 *	Geneva–Weiki	0.013	−0.201 *
	Weiki ^b^				
Temperature	30 °C ^a^	<0.001 *	30 °C–50 °C	0.013	−0.079 *
	50 °C ^b^				
Time	10 min ^a^	<0.001 *	10 min–60 min	0.018	−0.056 *
			10 min–30 min	0.018	−0.003
			10 min–180 min	0.017	−0.153 *
	30 min ^a^		60 min–30 min	0.018	0.053 *
	60 min ^b^		60 min–180 min	0.017	−0.097 *
	180 min ^c^		30 min–180 min	0.017	−0.151 *
Osmotic solution	Sucrose ^a^	<0.001 *	Xylitol–Maltitol	0.015	0.046 *
	Maltitol ^a^		Xylitol–Sucrose	0.015	0.045 *
	Xylitol ^b^		Maltitol–Sucrose	0.015	−0.007

Note: Means within columns, sharing the same superscript, are not significantly different from each other (Tukey’s HSD, *p* < 0.05). * denotes a statistically significant difference.

**Table 7:** Values of *RMSE*, *χ*^2^, *R*^2^ and *CRV* of best fitting mathematical model of SG.

Cultivar	Temperature	Solution	Model	*K*_1_	*K*_2_	*K*_s_	*RMSE*	*χ* ^2^	*CRV* (%)	*R* ^2^
Geneva	30 °C	sucrose	Ade-Omowaye	-	-	0.010	0.031	0.001	38.88	0.358
			Peleg	8.164	−0.001	-	0.018	0.000	22.76	0.780
		maltitol	Ade-Omowaye	-	-	0.044	0.043	0.002	14.16	0.961
			Peleg	1.021	0.024	-	0.038	0.001	12.54	0.969
		xylitol	Ade-Omowaye	-	-	0.024	0.049	0.003	36.22	0.832
			Peleg	−2.186	0.055	-	0.034	0.001	24.63	0.922
	50 °C	sucrose	Ade-Omowaye	-	-	0.023	0.023	0.001	16.42	0.940
			Peleg	1.137	0.055	-	0.026	0.001	18.86	0.921
		maltitol	Ade-Omowaye	-	-	0.022	0.027	0.001	19.37	0.873
			Peleg	3.339	0.038	-	0.024	0.001	17.29	0.898
		xylitol	Ade-Omowaye	-	-	0.029	0.043	0.002	23.76	0.872
			Peleg	2.103	0.016	-	0.038	0.002	21.20	0.898
Weiki	30 °C	sucrose	Ade-Omowaye	-	-	-	-	-	-	-
			Peleg	3.101	0.000	-	0.010	0.000	4.05	0.988
		maltitol	Ade-Omowaye	-	-	-	-	-	-	-
			Peleg	2.947	0.000	-	0.021	0.000	8.45	0.952
		xylitol	Ade-Omowaye	-	-	0.036	0.093	0.010	37.56	0.208
			Peleg	2.782	0.003	-	0.035	0.001	14.11	0.888
	50 °C	sucrose	Ade-Omowaye	-	-	0.044	0.121	0.018	39.43	0.066
			Peleg	2.266	0.002	-	0.029	0.001	9.63	0.944
		maltitol	Ade-Omowaye	-	-	0.044	0.099	0.012	33.64	0.431
			Peleg	2.244	0.012	-	0.051	0.003	17.36	0.848
		xylitol	Ade-Omowaye	-	-	0.052	0.101	0.012	29.18	0.626
			Peleg	1.871	0.032	-	0.078	0.007	22.59	0.776

“-” denotes values which cannot be calculated using this prediction model.

## Nomenclature

OD	osmotic dehydration,
WR	weight reduction, (%)
WL	water loss, (g/g i.d.m.)
SG	solid gain (g d.m./g i.d.m.)
m	sample mass, (g)
i.d.m.	initial dry matter, (g)
d.m.	dry solids mass of material, (g)
*τ*	time of osmotic dehydration, (min)
K_1_	parameter of Equation (7), (kg/kg)
K_2_	parameter of Equation (7), (kg/kg·h)
K*_ω_*	constant rate for water changes (4), (min^−0.5^)
K*_s_*	constant rate for solutes changes (5), (min^−0.5^)
K*_m_*	constant rate for solutes changes (6), (min^−0.5^)
Y	mean experimental value of WR, WL and SG respectively, (7)
MR_i,p_	predicted dimensionless moisture ratio,
MR_i,e_	experimental dimensionless moisture ratio,
MR_p_	experimental, mean dimensionless moisture ratio,
N	number of observation,
n	number of constants in the model equation,
Subscripts	
*o*	initial
*τ*	time (min)

## References

[1] Yadav A.K., Singh S.V. (2014). Osmotic dehydration of fruits and vegetables: A review. J. Food Sci. Technol..

[2] Chwastek A. (2014). Methods to increase the rate of mass transfer during osmotic dehydration of foods. Acta Sci. Pol. Technol. Aliment..

[3] Kowalski S.J., Mierzwa D. (2013). Influence of Osmotic Pretreatment on Kinetics of Convective Drying and Quality of Apples. Dry. Technol..

[4] Castelló M.L., Igual M., Fito P.J., Chiralt A. (2009). Influence of osmotic dehydration on texture, respiration and microbial stability of apple slices (Var. Granny Smith). J. Food Eng..

[5] Nuñez-Mancilla Y., Perez-Won M., Vega-Gálvez A., Arias V., Tabilo-Munizaga G., Briones-Labarca V., Lemus-Mondaca R., Di Scala K. (2011). Modeling mass transfer during osmotic dehydration of strawberries under high hydrostatic pressure conditions. Innov. Food Sci. Emerg. Technol..

[6] Held C., Sadowski G. (2016). Compatible solutes: Thermodynamic properties relevant for effective protection against osmotic stress. Fluid Phase Equilib..

[7] Held C., Neuhaus T., Sadowski G. (2010). Compatible solutes: Thermodynamic properties and biological impact of ectoines and prolines. Biophys. Chem..

[8] Rastogi N., Raghavarao K.S.M. (2004). Mass transfer during osmotic dehydration of pineapple: Considering Fickian diffusion in cubical configuration. LWT Food Sci. Technol..

[9] Megías-Pérez R., Gamboa-Santos J., Soria A.C., Villamiel M., Montilla A. (2014). Survey of quality indicators in commercial dehydrated fruits. Food Chem..

[10] United States Department of Agriculture Food Composition Databases Show Foods—Cranberries, Dried, Sweetened (Includes Foods for USDA’s Food Distribution Program). https://ndb.nal.usda.gov/ndb/foods/show/301093.

[11] Mendonça K.S., de Jesus J.R., Pereira M.C.A., Corrêa J.L.G. (2015). Osmotic Dehydration of Yacon Slices: Effect of Different Polyols on Mass Transfer Parameters.

[12] Venkatachalapathy K., Raghavan G.S.V. (1998). Microwave Drying of Osmotically Dehydrated Blueberries. J. Microw. Power Electromagn. Energy.

[13] Bialik M., Gondek E., Wiktor A., Latocha P., Witrowa-Rajchert D. (2017). Mathematical Modeling of *Actinidia arguta* (Kiwiberry) Drying Kinetics. Agric. Eng..

[14] Latocha P. (2017). The Nutritional and Health Benefits of Kiwiberry (*Actinidia arguta*)—A Review. Plant Foods Hum. Nutr..

[15] Latocha P., Łata B., Stasiak A. (2015). Phenolics, ascorbate and the antioxidant potential of kiwiberry vs. common kiwifruit: The effect of cultivar and tissue type. J. Funct. Foods.

[16] Rodrigues S., Fernandes F.A.N., Urwaye A.P. (2008). Ultrasound in food processing. New Food Engineering Research Trends.

[17] Tiroutchelvame D., Sivakumar V., Maran P. (2015). Mass transfer kinetics during osmotic dehydration of amla (*Emblica officinalis* L.) cubes in sugar solution. Chem. Ind. Chem. Eng. Q..

[18] Arballo J.R., Bambicha R.R., Campañone L.A., Agnelli M.E., Mascheroni R.H. (2012). Mass transfer kinetics and regressional-desirability optimisation during osmotic dehydration of pumpkin, kiwi and pear: Kinetics/optimisation OD-pumpkin, kiwi, pear. Int. J. Food Sci. Technol..

[19] Alam M.S., Amarjit S., Sawhney B.K. (2010). Response surface optimization of osmotic dehydration process for aonla slices. J. Food Sci. Technol..

[20] Yadav B.S., Yadav R.B., Jatain M. (2012). Optimization of osmotic dehydration conditions of peach slices in sucrose solution using response surface methodology. J. Food Sci. Technol..

[21] Cichowska J., Żubernik J., Czyżewski J., Kowalska H., Witrowa-Rajchert D. (2018). Efficiency of Osmotic Dehydration of Apples in Polyols Solutions. Molecules.

[22] Koocheki A., Azarpazhooh E. (2010). Evaluation of Mass Exchange during Osmotic Dehydration of Plum Using Response Surface Methodology. Int. J. Food Prop..

[23] Cao H., Zhang M., Mujumdar A.S., Du W., Sun J. (2006). Optimization of Osmotic Dehydration of Kiwifruit. Dry. Technol..

[24] Peleg M. (1988). An Empirical Model for the Description of Moisture Sorption Curves. J. Food Sci..

[25] Ade-Omowaye B.I.O., Rastogi N.K., Angersbach A., Knorr D. (2003). Combined effects of pulsed electric field pre-treatment and partial osmotic dehydration on air drying behaviour of red bell pepper. J. Food Eng..

[26] Wiktor A., Śledź M., Nowacka M., Chudoba T., Witrowa-Rajchert D. (2014). Pulsed Electric Field Pretreatment for Osmotic Dehydration of Apple Tissue: Experimental and Mathematical Modeling Studies. Dry. Technol..

[27] Torreggiani D. (1993). Osmotic dehydration in fruit and vegetable processing. Food Res. Int..

[28] Assis F.R., Morais R.M.S.C., Morais A.M.M.B. (2016). Mass Transfer in Osmotic Dehydration of Food Products: Comparison between Mathematical Models. Food Eng. Rev..

[29] Panagiotou N.M., Karathanos V.T., Maroulis Z.B. (1998). Mass transfer modelling of the osmotic dehydration of some fruits: Osmotic dehydration of fruits. Int. J. Food Sci. Technol..

[30] Ciurzyńska A., Cichowska J., Kowalska H., Czajkowska K., Lenart A. (2018). Osmotic dehydration of Braeburn variety apples in the production of sustainable food products. Int. Agrophys..

[31] Ganjloo A., Rahman R.A., Bakar J., Osman A., Bimakr M. (2012). Kinetics Modeling of Mass Transfer Using Peleg’s Equation During Osmotic Dehydration of Seedless Guava (*Psidium guajava* L.): Effect of Process Parameters. Food Bioprocess Technol..

[32] Chiu M.-T., Tham H.J., Lee J.-S. (2017). Optimization of osmotic dehydration of *Terung Asam* (*Solanum lasiocarpum* Dunal). J. Food Sci. Technol..

[33] Lee J.-S., Tham H.J., Wong C.S. (2014). Osmotic dehydration of Kappaphycus alvarezii. J. Appl. Phycol..

[34] Lazarides H.N., Katsanidis E., Nickolaidis A. (1995). Mass transfer kinetics during osmotic preconcentration aiming at minimal solid uptake. J. Food Eng..

[35] Latimer G.W., AOAC International (2016). Official Methods of Analysis of AOAC International.

